# Association between HLA Class I Alleles and Proviral Load in HTLV-I Associated Myelopathy/Tropical Spastic Paraperesis (HAM/TSP) Patients in Iranian Population

**Published:** 2013-03

**Authors:** Mahdi Taghaddosi, S. A. Rahim Rezaee, Houshang Rafatpanah, Taraneh Rajaei, Reza Farid Hosseini, Valizadeh Narges

**Affiliations:** 1 HTLV-I Foundation, Ghaem Hospital, Mashhad University of Medical Sciences, Mashhad, Iran; 2 Immunology Research Centre, Faculty of Medicine, Mashhad University of Medical Sciences, Mashhad, Iran; 3 Inflammation and inflammatory Disease Research Centre, Faculty of Medicine ,Mashhad University of Medical Sciences, Mashhad, Iran; 4 Allergy Research Centre, School of Medicine, Mashhad University of Medical Sciences, Mashhad, Iran

**Keywords:** HTLV-I, HAM/TSP, HLA, Proviral load

## Abstract

***Objective(s):*** The aim of this study was to investigate the association between HLA class I alleles (HLA-A*02, HLA-A*24, HLA-Cw*08, HLA-B5401) and proviral load in HTLV-I associated myelopathy/tropical spastic paraperesis (HAM/TSP) patients in Iranian population.

***Materials and Methods:*** 20 new cases of HAM/TSP patients and 30 HTLV-I infected healthy carriers were recruited. Peripheral blood samples were collected. Peripheral blood mononuclear cells (PBMCs) were isolated. DNA was extracted from PBMC.HTLV-I proviral load was calculated by Taqman quantitative real time polymerase chain reaction (qRT-PCR). PCR sequence-speciﬁc primer (PCR-SSP) reactions were performed to detect HLA-A, HLA-B and, HLA-Cw alleles.

***Results:*** There was no signiﬁcant difference in sex and age between asymptomatic and HAM/TSP group. The Mann-Whitney U test was used to compare proviral load between HAM/TSP patients and healthy carrier. Provirus load of HAM/TSP patients was signiﬁcantly higher than that of HCs (*P=*0.003, Mann–Whitney U test).Odd ratio was calculated to determine association between class I alleles including (HLA-A*02, HLA-A*24, HLA-Cw*08) and risk of HAM/TSP development. We couldn’t find any association between these class I alleles and risk of HAM/TSP development in our study. In our survey HLA-A*02, HLA-A24, HLA-Cw*08 didn’t have protective effect on proviral load (*P=*0.075, *P=*0.060 and 0.650 Mann–Whitney U test respectively).

***Conclusion:*** In conclusion, certain HLA alleles with protective effect in one population may have not similar effect in other population. This may be because of pathogen polymorphism or host genetic heterogeneity and allele frequency in desired population.

## Introduction

Human T-lymphotropic virus type1 (HTLV-I) was the first human retrovirus discovered in 1977 (1). It has been demonstrated to be etiological agent in adult T cell leukemia (ATL) and a progressive neurological disease called HTLV-I associated myelopathy/tropical spastic paraparesis (HAM/TSP) (2-3).This neurological disease manifested as a progressive and slow spastic paraparesis without remission(4). The vast majority of HTLV-I-infected individuals are clinically asymptomatic; less than 5% of infected individuals develop HAM/TSP (5). Although the risk factors causing different manifestations of HTLV-I infection are not fully understood, the pathogenesis of HTLV-I-associated diseases is thought to be in part due to proviral load or virus-infected cell burden variations (6-7). The population association studies suggested that both viral and host genetic factors influence the outcome of infection (8-9). Human leukocyte antigen (HLA) class I genes, have been associated with outcome in many human infections and it is among host genetic factors that have effect on manifestation of HAM/TSP. Speciﬁc HLA alleles have been linked to protection from developing HAM/TSP, whereas other HLA alleles have been correlated with an increased risk of developing HAM/TSP (9-10). The viral protein Tax, encoded within the pX region, has been demonstrated to be a critical factor for host genomic activation and viral gene expression(11). Studies have indicated that the HLA-A*02 allele has a protective role in HAM/TSP disease (12). It has been suggested that a strong antiviral cytotoxic T lymphocytes (CTL) response is initiated by individuals with this HLA type by virtue of its high binding affinity to the Tax (residues 11–19) peptide, resulting in targeted killing of HTLV-I-infected cells and a subsequent low proviral DNA load, thus can protect the infected subject from HAM/TSP. HLA-Cw*08 allele is independently and signiﬁcantly associated with a lower proviral load and a lower risk of HAM/TSP (12). Furthermore, in vitro analysis demonstrated that tax301-309 peptide binds strongly to HLA-A*24 and reduces proviral load(13).In contrast, HLA-B*5401 is associated with an increased susceptibility to HAM/TSP (10). In this study association between HLA class I allele including: HLA-A*02, HLA-Cw*08, HLA-B5401 and HLA-A*24 and proviral load were investigated in northeastern Iran, an endemic area of HTLV-Iin Middle East (9).

## Materials and Methods

*Patients*

Peripheral blood samples were obtained from 20 new cases of HAM/TSP patients who did not receive any medication and 30 healthy carrier from blood donors of the Blood Transfusion Centre, the headquarter of Khorasan Razavi. The study was approved in medical ethic committee (project code: 900183) of Mashhad University of medical sciences (MUMS). An informed consent was obtained from all individuals. The diagnosis of HAM/TSP was made in accordance with World Health Organization criteria (14). HTLV-I infection was confirmed by PCR. Peripheral blood mononuclear cells (PBMCs) were isolated from EDTA-treated blood samples by a Histopaque-1077 (Sigma, Germany) density gradient. DNA was extracted using an available commercial kit (DNA blood mini kit, Qiagen Germany).

*HTLV-I proviral load assay*

To assess the HTLV-I proviral load, a Taqman quantitative real time polymerase chain reaction (qRT-PCR) was performed using an available commercial kit (Novin Gene, Iran). In short, a Taqman quantitative real time PCR method was carried out to measure the proviral load of HTLV-I in PBMCs by a corbet Rotorgen Q (Qiagen, Germany) Real-Time PCR machine. The HTLV-I copy number was calculated by comparing with the quantification of the albumin gene as the reference gene. HTLV-I and albumin DNA concentrations were calculated using standard curves. The HTLV-I proviral load was calculated as the ratio of HTLV-I DNA copies number / albumin DNA copies number / 2 × 10^4^ and expressed as the number of HTLV-I proviruses per 10^4^ PBMCs (15).

*HLA typing*

PCR sequence-speciﬁc primer(PCR-SSP) reactions were performed to detect HLA-A, HLA-B and, HLA-Cw(16). HLA-A, HLA -B and HLA-Cw were typed to two digits by PCR-SSP method. The kits Texas BioGene ABC SSP Tray (Texas BioGene, Taiwan,) were used to amplify exons 2 & 3 of HLA-A, HLA-B and HLA-C loci according to the manufacturer’s instruction. PCR products were separated by electrophoresis on a 2% agarose gel stained with ethidium bromide and evaluated under UV light. The sizes of PCR products were estimated according to the migration pattern of a 100-bp DNA ladder. All the gels were documented and the pattern of positive bands was analyzed using software (Morgan™ SSPal HLA Typing Analysis Software**).**

## Results

A total of 50 samples (HAM/TSP group: 20 subjects, Carrier group: 30 subjects) were analyzed. There was no signiﬁcant difference in sex and age between asymptomatic and HAM/TSP group. The HTLV-I DNA copy mean in asymptomatic carrier was 206.0±85 per 10^4 ^PBMC and in HAM/TSP patient was 677±14. Provirus load of HAM/TSP patients was signiﬁcantly higher than that of HCs (*P=*0.003, Mann–Whitney U test).

To examine association between HLA class I alleles and HAM/TSP HLA-typing was performed by PCR-SSP method to determine HLA-A*02, HLA-Cw*08, HLA-A*24 and HLA-B*54 in 50 HTLV-I infected individuals (20 HAM/TSP and 30 asymptomatic carriers).we couldn’t find any association between these class I alleles including (HLA-A*02, HLA-A*24, HLA-Cw*08) and risk of HAM/TSP development.

**Figure 1:** Proviral load in HAM/TSP and healthy carrier

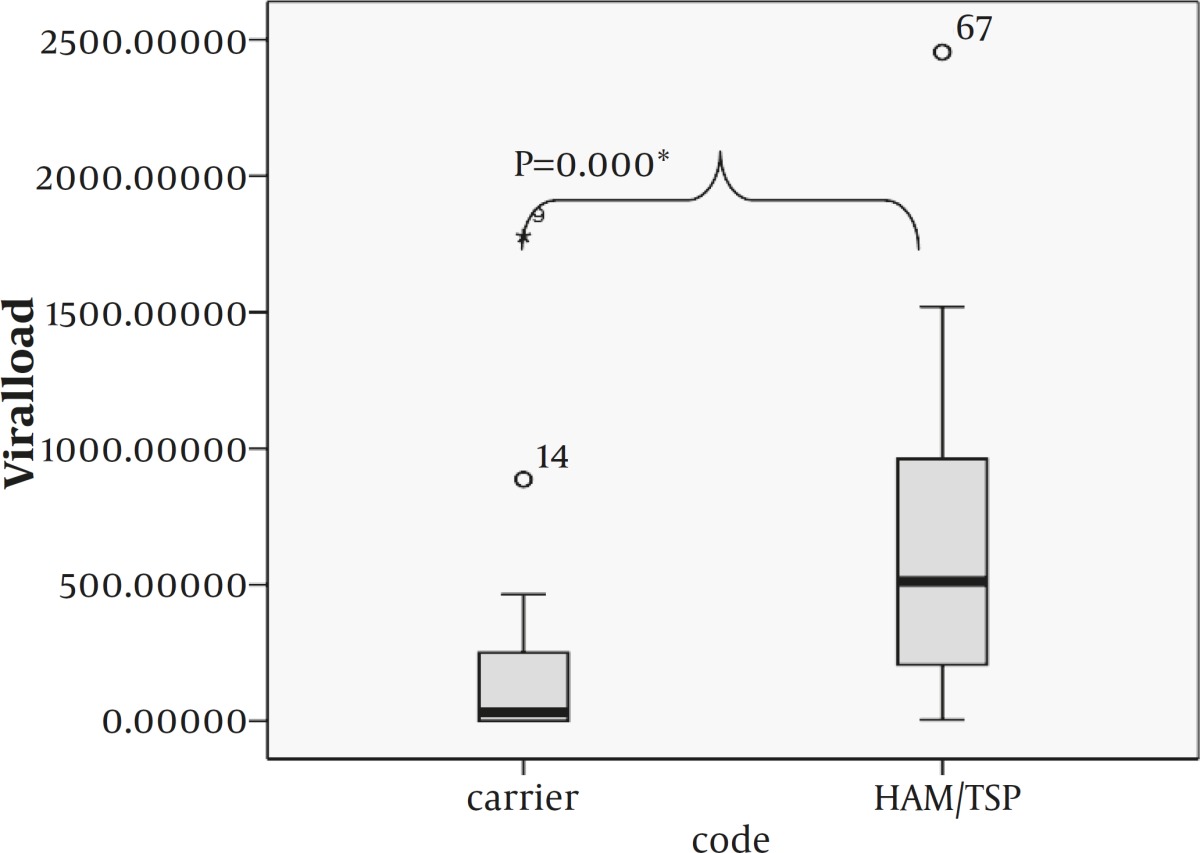

HLA-A*02, HLA-Cw*08 and HLA-A*24 are not associated with a lower risk of HAM/TSP and a lower provirus load in Iranian HTLV-I-infected

**Table 1:** HLA-A*02, HLA-Cw*08 and HLA-A24 were not associated with a lower risk of HAM/TSP

HLA allele	HAM/TSP	Healthy carrier	OR
HLA-A*02^+^	*6*	*7*	*1.40*
HLA-A*02^- ^	*14*	*23*	
HLA-Cw*08^+^	*3*	*4*	*1.14*
HLA-Cw*08^- ^	*17*	*26*	
HLA-A*24^+^	*6*	*8*	*1.18*
HLA-A*24^-^	*14*	*22*	

In our survey HLA-A*02, HLA-A24, HLA-Cw*08 didn’t have protective effect on proviral load (*P=*0.075, *P=*0.060 and 0.650 Mann–Whitney U test respectively). Also we couldn’t find HLA-B*5401 in our study population, because this allele is not prevalent in Iranian population. It is known to be almost exclusively found in East Asian populations.

## Discussion

The differences between individuals in the quality and quantity of the immune response to a given pathogen largely depend on polymorphism in genes that control the immune response (17). The list of candidate gene polymorphisms is long. However, the human leukocyte antigen (HLA) is the most important group of candidate genes, because of their central role in the immune response. The power of candidate gene studies is limited by the choice of candidate genes, whereas the effectiveness of genome-wide searches for genetic determinants is affected by the low statistical power after correction for multiple statistical tests(8). In this study, we analyzed the effect of HLA class I alleles including HLA-A*02, HLA-Cw*08, HLA-B*5401 and HLA-A*24 on HTLV-I proviral load and risk of HAM/TSP development. HTLV-I Tax301-309 is newly identified as an immunodominant epitope restricted to HLA-A24. It has been demonstrated that the frequency of HTLV-I Tax301-309-specific CTLs is higher in HAM/TSP patients than in ACs and negatively correlated with the HTLV-I proviral load in both HAM/TSP patients and ACs (13). We have analyzed the effect of HLA-A24 allele on HTLV-I proviral load and risk of HAM/TSP development in our study but we couldn’t find any correlation between this allele and proviral load (*P=*0.060). Kagoshima cohort of HAM/TSP is the world’s largest study which surveys the effect of HLA genes on HAM/TSP development. In the Kagoshima population, an association between HLA-B*5401, HLA-A*02 and HLA-Cw*08 and the outcome of HTLV-I infection has been reported, where HLA-A*02and HLA-Cw*08 genes were each independently associated with a lower HTLV-I provirus load and with protection from HAM/TSP, whereas HLA-B*5401 was associated with an increased susceptibility to HAM/TSP (10). HLA-A*02 is a common allele in our population and elsewhere. We believe that this is why it has been possible to estimate the effect of this allele in this survey. In our study we couldn’t find any correlation between HLA-A*02, HLA-Cw*08, (*P=*0.075, *P=*0.650 irrespectively) and HTLV-I proviral load. There are signiﬁcant differences between populations in the genetic contribution to susceptibility to HAM/TSP, since HLA-B*5401 is frequent allele in Japan and in East Asian populations, but it is rare in other populations including Iran (18). We couldn’t find this allele in our asymptomatic carrier and HAM/TSP patients. The main cause for this discrepancy between our study and previous analysis is attributable to differences between HLA allele frequency in different population. Our finding suggests that frequency of certain HLA class I allele, which has important effect on outcome of HTLV-I infection, is one of the most crucial factors in defining the outcome of infection in different ethnic population. Also geographical variation in pathogen polymorphism is superimposed on this host genetic heterogeneity (19). The best example is HLA-A*02 allele which binds with different strength to different Tax protein variants (20). According to this background, the most practical approach to survey the effect of certain HLA allele in development of HAM/TSP is to consider its frequency in desired population. Certain HLA alleles with protective effect in one population may have not similar effect in other population. This may be because of pathogen polymorphism or host genetic heterogeneity.
